# A Potential Inhibitory Profile of Liver CD68^+^ Cells during HCV Infection as Observed by an Increased CD80 and PD-L1 but Not CD86 Expression

**DOI:** 10.1371/journal.pone.0153191

**Published:** 2016-04-11

**Authors:** Elias A. Said, Iman Al-Reesi, Marwa Al-Riyami, Khalid Al-Naamani, Shadia Al-Sinawi, Mohammed S. Al-Balushi, Crystal Y. Koh, Juma Z. Al-Busaidi, Mohamed A. Idris, Ali A. Al-Jabri

**Affiliations:** 1 Department of Microbiology and Immunology, College of Medicine and Health Sciences, Sultan Qaboos University, P.O. Box: 35, Code: 123, Muscat, Oman; 2 Department of Pathology, College of Medicine and Health Sciences, Sultan Qaboos University, P.O. Box: 35, Code: 123, Muscat, Oman; 3 Department of Medicine, Armed Forces Hospital, Muscat, Oman; Institut Pasteur, FRANCE

## Abstract

**Aim:**

The lack of potent innate immune responses during HCV infection might lead to a delay in initiating adaptive immune responses. Kupffer cells (KCs) and liver-infiltrating monocytes/macrophages (CD68^+^ cells) are essential to establish effective anti-HCV responses. They express co-stimulatory molecules, CD80 and CD86. CD86 upregulation induces activator responses that are then potentially regulated by CD80. The relative levels of expression of CD80, CD86 and the inhibitory molecule, PD-L1, on CD68^+^ cells modulate T cell activation. A few studies have explored CD80 and PD-L1 expression on KCs and infiltrating monocytes/macrophages in HCV-infected livers, and none investigated CD86 expression in these cells. These studies have identified these cells based on morphology only. We investigated the stimulatory/inhibitory profile of CD68^+^ cells in HCV-infected livers based on the balance of CD80, CD86 and PD-L1 expression.

**Methods:**

CD80, CD86 and PD-L1 expression by CD68^+^ cells in the lobular and portal areas of the liver of chronic HCV-infected (n = 16) and control (n = 14) individuals was investigated using double staining immunohistochemistry.

**Results:**

The count of CD68^+^ KCs in the lobular areas of the HCV-infected livers was lower than that in the control (*p* = 0.041). The frequencies of CD68^+^CD80^+^ cells and CD68^+^PD-L1^+^ cells in both lobular and total areas of the liver were higher in HCV-infected patients compared with those in the control group (*p* = 0.001, 0.031 and 0.007 respectively). Moreover, in the lobular areas of the HCV-infected livers, the frequency of CD68^+^CD80^+^ cells was higher than that of CD68^+^CD86^+^ and CD68^+^PD-L1^+^ cells. In addition, the frequencies of CD68^+^CD80^+^ and CD68^+^CD86^+^ cells were higher in the lobular areas than the portal areas.

**Conclusions:**

Our results show that CD68^+^ cells have an inhibitory profile in the HCV-infected livers. This might help explain the delayed T cell response and viral persistence during HCV infection.

## Introduction

More than 185 million people around the world are infected with hepatitis C virus (HCV)[1]. HCV infection causes liver inflammation, and can lead to fibrosis/cirrhosis and hepatocellular carcinoma[2]. Controlling HCV infection and its outcome depends on the efficacy of the immune response, which is regulated by the interaction between the components of the innate and adaptive immune system mainly in the liver[2]. The adaptive immune response during HCV infection is generally delayed, irrespective of the disease progression and outcome suggesting a lack of suitable innate immune responses[3,4]. The main population of innate immune cells in the liver is constituted of macrophages residing in the liver and known as Kupffer cells (KCs) and infiltrating monocytes/macrophages[2]. KCs and liver-infiltrating macrophages play an important role in the immune activation, antiviral immunity and tissue damage associated with HCV infection[2].

CD80 (B7.1) and CD86 (B7.2) are the main co-stimulatory molecules expressed by KCs and infiltrating macrophages in the liver. These molecules participate in regulating T cell responses[5]. Both CD80 and CD86 interact with CD28 expressed on T cells to deliver an activating signal, and with cytotoxic T-lymphocyte-associated protein 4 (CTLA-4), which competes with CD28, to deliver an inhibitory signal[5]. Although CD80 and CD86 seem to have redundant functions, CD80 is upregulated on antigen presenting cells (APCs) later than CD86 at a time when CTLA-4 is already upregulated on T cells. CD80 has a greater capacity to induce inhibitory signals, through its interaction with CTLA-4, than CD86[6,7,8]. Moreover, CTLA-4 has a high capacity to deplete CD80 from the surface of APCs, thus preventing its interaction with CD28 to deliver stimulatory signals[9,10]. Therefore, it is possible that the upregulation of CD86 is prompt to induce activator responses, while CD80 expression regulates the subsequent responses[7]. On the other hand, programmed cell death-1 (PD-1) ligands 1 and 2 (PD-L1 and PD-L2) are expressed by APCs including KCs and infiltrating monocytes/macrophages to prevent unnecessary activation and hyper-activation and avoid tissue damage caused by activated T cells[11]. Relative levels of the inhibitory PD-L1 signal and co-stimulatory CD80/CD86 signals on APCs might determine the extent of T cell activation and the threshold between tolerance and autoimmunity[12]. Although the role of KCs in HCV pathogenesis is still poorly understood, changes in the frequency and level of activation of KCs and liver-infiltrating macrophages during HCV infection have been reported. Some studies reported that type I IFN production by KCs is suppressed by HCV and that elevated IL-10 production was found in KCs, which in turn suppresses pro-inflammatory cytokine production by intrahepatic cells and disturbs antigen presentation to T cells[2]. Moreover, a few studies investigating the expression of CD80 and PD-L1 on KCs during HCV infection have shown that these molecules are upregulated on KCs in HCV-infected patients[13,14]. However, these studies identified KCs based on their morphology alone, and the expression of CD80, CD86 and PD-L1 together was not investigated in the same patient. To our knowledge, no previous study has investigated the expression of CD86 on KCs and infiltrating monocytes/macrophages during HCV infection.

Human monocytes/macrophages and KCs can be identified by immunohistochemistry or flow cytometry using antibodies directed against CD68, CD163, CD14 and CD16[2]. However, the levels of CD163, CD14 and CD16 can be modulated by stimulation[15,16]. This study is the first to use a double staining immunohistochemistry (IHC) method to investigate the differences in the expression of CD80, CD86 and PD-L1 in CD68^+^ cells in the lobular and portal areas of the liver during HCV infection. This could help in understanding the potential stimulatory/inhibitory profile of monocytes/macrophages and KCs in the liver based on the balance in the expression of these molecules in HCV-infected patients.

## Materials and Methods

### Study population

Formalin fixed paraffin embedded specimens of liver tissue were obtained from 16 patients with chronic HCV infection and 14 virus-negative individuals as a control group (Table 1). All specimens were obtained from the Pathology Departments at the Sultan Qaboos University Hospital (SQUH) and the Armed Force Hospital (AFH), Muscat, Oman. Individuals with autoimmune diseases, microbial infections other than HCV and patients on anti-HCV therapy, active ethanol abuse, non-alcoholic steatohepatitis, metabolic liver diseases, drug/toxin-induced hepatitis or patients with established cirrhosis were excluded. All HCV-infected patients were positive for anti-HCV and had detectable HCV RNA. The control group included individuals with high levels of liver enzymes of unknown etiology and liver transplant donors. Biopsies of unaffected areas from resection specimens of hepatocellular carcinoma were also included in the control group. The same control group was used in a study, which was conducted in parallel, about the expression of CD80, CD86 and PD-L1 in liver CD68^+^ cells during chronic HBV infection. Because the experimental procedure consisted of staining that was performed on biopsies that were already done and stored in the bank of the Pathology Department, and did not involve any special sample (biopsy) collection (article 32 of the Declaration of Helsinki), patients consent was impracticable to obtain. The study and the procedure (the absence of consent) were approved by the Sultan Qaboos University Ethics Committee (MREC#742). The data were analyzed anonymously. To maintain confidentiality, every patient was assigned with a unique identification number.

**Table 1:** Characteristics of the study population. Chronic HCV-infected patients (n = 16) and Control individuals (n = 14) were included in the study.

	Gender (M/F)	Mean age (Yr)	VL (x10^5^IU/ml)
**Control group**	9/5	30.5±9.7	-
**HCV-infected patients**	7/9	35.5±7.1	20.2 ± 32.7

M = male; F = female; Yr = Year; VL = viral load

### Immunohistochemistry (IHC) and identification of cells

KCs and liver-infiltrating macrophages/monocytes were identified based on their morphology and expression of CD68. The liver sections were double-stained to detect the expression of PD-L1, CD80 or CD86 using the double staining kit [Polink DS-MR-Hu D2 Kit, IHC, Golden Bridge International Lab (GBI); US] following the manufacturer’s protocol. Heat induced epitope retrieval (HIER) was performed using an antigen retrieval buffer at pH 9.0 for 20 minutes (min). Optimal dilutions of the primary antibodies were established by titrating on tonsillar tissue. For each set of staining, a section of tonsil was included as a positive control whereas a negative control consisted of replacing primary antibodies (Abs) with an isotype Ab or Ab diluent. Nonspecific proteins were blocked, and then primary Abs were added for 30 min at room temperature and washed with Tris-buffered saline. Alkaline phosphatase (AP) polymer anti-mouse IgG was used to detect monoclonal mouse anti-CD68 Ab (Dako, USA) and horseradish peroxidase (HRP) polymer anti-rabbit IgG was used to detect rabbit anti-CD80, CD86 or PD-L1 Abs (Abcam, UK). Two distinct chromogens, GBI Permanent-Red (red color, used with AP polymer anti-mouse IgG) and Emerald chromogen (green color, used with HRP polymer anti-Rabbit IgG), were used. Slides were examined using light microscopy (Olympus BX53 microscope). CD68^+^cells stained red and the co-localization was indicated by a presence of both green (PD-L1, CD80 or CD86) and red (CD68) colors in the same cell. Positive cells were counted in five high power fields (diameter of the field = 0.44 mm) with 60x objective and 10x ocular lenses. This was done for both the portal and lobular areas. Hematoxylin and Eosin and Masson Trichrome stained slides were used to assess the morphology of the liver biopsy. Positive cell counting was done by two independent expert/trained readers i.e. M.R. (Department of Pathology) and I.R. (Department of Microbiology and Immunology).

### Statistical analysis

Mann-Whitney test was used to assess the significance of the differences in the cell count and protein expression in the lobular and portal areas of the liver between the HCV-infected patients and the controls. Wilcoxon test was used to assess the significance of the differences in the expression of the three proteins within the same group or between the two areas of the liver in each subject (paired). A *p* value < 0.05 was considered statistically significant. Statistical Package for the Social Sciences (SPSS) version 20, Microsoft Excel and Prism softwares were used to analyze the results.

## Results

### HCV-infected patients have low CD68^+^ cell counts within the lobular areas of the liver

**Fig 1:**
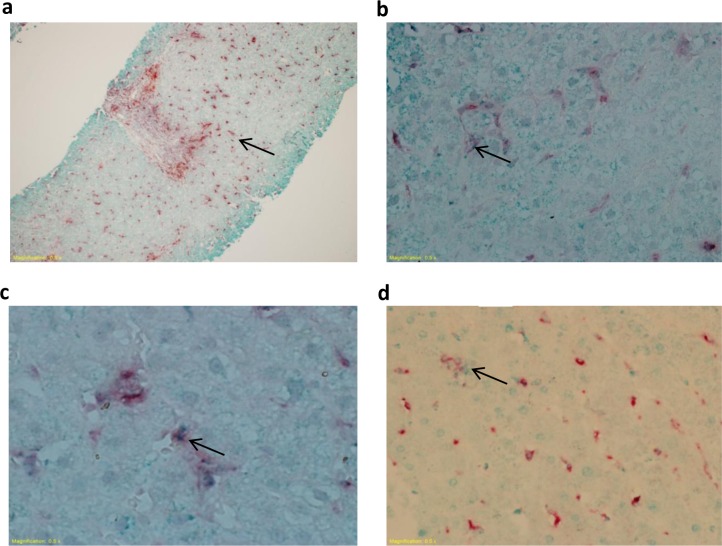
CD68, CD80, CD86 and PD-L1 staining in liver biopsies. Biopsies from HCV-infected and control individuals were stained with mouse anti-human CD68 and rabbit anti-human CD80, CD86 or PD-L1 Abs. Representative photomicrographs showing CD68 red staining indicating CD68^+^ cells (**A**) and CD80, CD86 or PD-L1 green staining (**B-D**) observed as dots (indicated by the arrows) within the red stained cells indicating CD68^+^CD80^+^, CD68^+^CD86^+^ and CD68^+^PD-L1^+^ cells. This figure is also presented in a related article about the expression of CD80, CD86 and PD-L1 in liver CD68^+^ cells during chronic HBV infection as the same control group was used in both studies that were conducted in parallel.

Within the hepatic lobules, KCs took the form of spindle like cells lining the sinusoids, and in the portal areas monocytes/macrophages appeared as round cells. We have used a double staining IHC technique, which combined anti-CD68 Ab with anti-CD80, anti-CD86 or anti-PD-L1 Abs. CD68^+^ cells in the lobular and portal areas stained red indicating CD68 expression (Fig 1A). CD80, CD86 or PD-L1 staining was observed as small green dots within the red stained cells (Fig 1B, 1C and 1D respectively).

**Fig 2:**
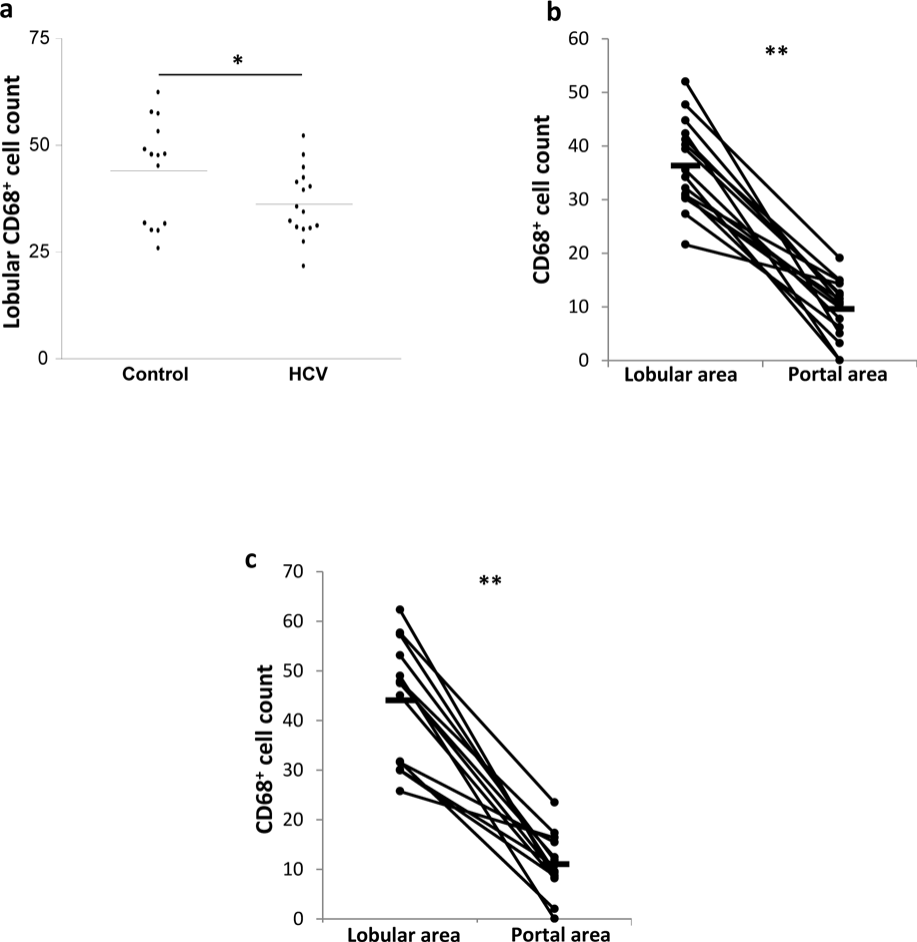
HCV infection is associated with low CD68^+^ cell count in the lobular areas of the liver. Biopsies from HCV-infected and control individuals were stained with mouse anti-human CD68 Ab. **A.** CD68^+^ cell count in the lobular areas of the liver of control and HCV-infected individuals. **B.** CD68^+^ cell count in the lobular and portal areas of HCV-infected livers. **C.** CD68^+^ cell count in the lobular and portal areas of control livers. * *P* value<0.05. ** *P* value<0.01.

To determine whether HCV infection affects the number of CD68^+^ cells in the liver, the counts of CD68^+^ cells were obtained based on the staining with the anti-CD68 Ab. The KC count in the lobular areas was significantly lower in the HCV-infected patients than in the controls (1.3-fold; *p* = 0.041; Fig 2A). In contrast, neither CD68^+^ cell counts in the portal areas nor the total counts of CD68^+^ cells (the portal and lobular areas combined) were significantly different between the HCV-infected patients and the controls (Table 2).

**Table 2:** Cell counts based on the expression of CD68 alone or together with CD80, CD86 or PD-L1. The average counts ± S.D. are represented for each areas as indicated. The *p* values were calculated to assess the significance of differences between the HCV-infected and control group.

	Lobular	Portal	Total
	**CD68**^**+**^ **count**		
**HCV-infected patients**	36.3 ± 9.5	9.5 ± 5.5	45.8 ± 9.8
**Controls**	44 ± 12	10.9 ± 6	54.9 ± 13.8
***P* value**	0.041	0.575	0.244
	**CD68**^**+**^**CD80**^**+**^ **count**		
**HCV-infected patients**	17.9 ± 14.2	5.3 ± 9.2	23.3 ± 20.3
**Controls**	7.6 ± 11.5	1.8 ± 2.8	9.3 ± 11.9
***P* value**	0.002	0.332	0.015
	**CD68**^**+**^**CD86**^**+**^ **count**		
**HCV-infected patients**	7.5 ± 12.5	2.8 ± 6.9	10.2 ± 19.8
**Controls**	3.9 ± 3.9	1.6 ± 2.3	5.5 ± 5.7
***P* value**	0.382	0.344	0.467
	**CD68**^**+**^**PD-L1**^**+**^ **count**		
**HCV-infected patients**	8.4 ± 9.1	1.4 ± 1.12	10.8 ± 9.8
**Controls**	2.5 ± 2	0.5 ± 0.8	2.9 ± 2.6
***P* value**	0.07	0.058	0.028

Although monocytes/macrophages are present throughout the liver, their density differs between the areas of the liver[17]. To determine the effect of chronic HCV infection on the distribution of CD68^+^ cells between the portal and the lobular areas of the liver, the counts of CD68^+^ cells in these areas were compared within each group. The CD68^+^ cell count was ≈3-fold higher in the lobular areas than the portal areas in both the patients and the controls (*p* = 0.004 and *p* = 0.006; Fig 2B and 2C).

The CD68^**+**^ cell count did not correlate with HCV viral load (data not shown). Moreover, we did not observe any association between the counts of CD68^+^ cells and the age and sex of the individuals in the HCV-infected or the control groups (data not shown).

### An increase in the CD68^+^CD80^+^ cell frequency in the lobular areas of the liver of the HCV-infected patients

**Fig 3:**
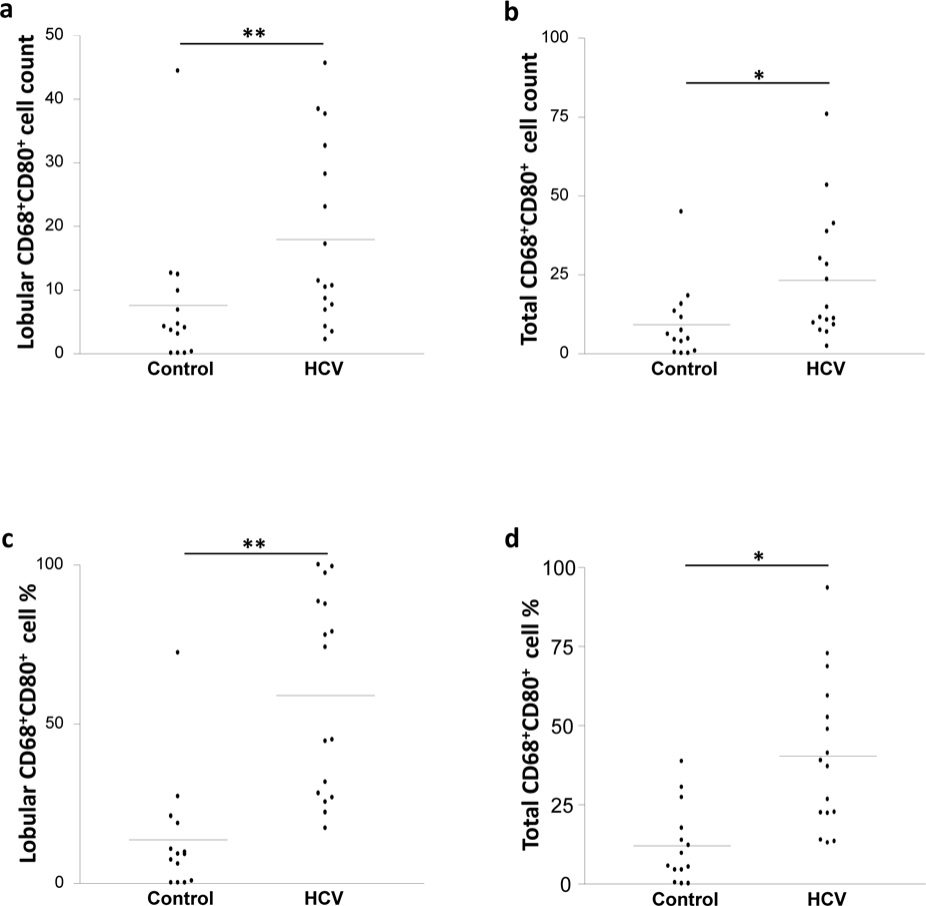
HCV infection is associated with elevated CD68^+^CD80^+^ cell frequency in the lobular areas of the liver. Biopsies from HCV-infected and control individuals were stained with mouse anti-human CD68 and rabbit anti-human CD80 Abs. CD68^+^CD80^+^ cell count in (**A**) the lobular areas and (**B**) the total (lobular and portal) areas of the liver of control and HCV-infected individuals. CD68^+^CD80^+^ cell percentage in (**C**) the lobular areas and (**D**) the total (lobular and portal) areas of the liver of control and HCV-infected individuals. * *P* value<0.05. ** *P* value<0.01.

The capacity of macrophages to control T cell stimulation depends on the co-stimulatory signals they deliver to T cells. We investigated the expression of the co-stimulatory molecules, CD80 and CD86 on CD68^+^ cells using double staining based IHC (anti-CD68 Ab with anti-CD80 or anti-CD86 Abs). The count of CD68^+^CD80^+^ KCs in the lobular areas of the liver was 2-fold higher in the HCV-infected patients than the controls (*p* = 0.002; Fig 3A) while the cell counts in the portal areas were not significantly different between the two groups (Table 2). When the total counts of CD68^+^CD80^+^ cells in the lobular and portal areas together were considered, the HCV-infected patients showed a 2-fold increase over the control group (*p* = 0.015; Fig 3B).

We also examined the percentages of CD80^+^ and CD86^+^ cells among CD68^+^ cells present in the lobular and portal areas of the liver separately as well as combined. HCV-infected patients showed a 4-fold increase in the percentage of CD68^+^CD80^+^ KCs in the lobular areas, but not portal areas, compared with the controls *(p* = 0.001; Fig 3C). When the total percentages (lobular and portal areas together) were considered, HCV-infected patients showed a 3-fold increase compared with the controls (*p* = 0.01; Fig 3D).

In contrast, no significant differences were found in the count and percentage of CD68^+^CD86^+^ cells in the lobular, portal and total areas when comparing HCV-infected patients with the controls (Tables 2 and 3).

**Table 3:** Cell percentage based on the expression of CD68 together with CD80, CD86 or PD-L1. The average of the percentages ± S.D. are presented for each areas as indicated. The *p* value was calculated.

	Lobular	Portal	Total
**CD68**^**+**^**CD80**^**+**^ **Frequency**			
**HCV-infected patients**	59.1 ± 31.5	26.5 ± 25.7	40.5 ± 23.9
**Controls**	13.7 ± 18.8	12.9 ± 15.8	12.1 ± 12.3
***P* value**	0.001	0.525	0.01
**CD68**^**+**^**CD86**^**+**^ **Frequency**			
**HCV-infected patients**	14.7 ± 13.9	7.2 ± 10.8	21.6 ± 20.7
**Controls**	9.2 ± 10.6	10.5 ± 12.2	14.3 ± 15.4
***P* value**	0.212	0.209	0.708
**CD68**^**+**^**PD-L1**^**+**^ **Frequency**			
**HCV-infected patients**	22.1 ± 25.1	11.8 ± 12.4	31.9 ± 27.6
**Controls**	6.4 ± 5.6	3.9 ± 4.6	10.5 ± 8.8
***P* value**	0.031	0.147	0.007

### An increase in CD68^+^PD-L1^+^ cell frequency in the liver of the HCV-infected patients

Since we were interested in understanding the potential stimulatory/inhibitory profile of CD68^+^ cells in the liver of the studied population, the expression of PD-L1 was also assessed using a combination of anti-CD68 and anti-PD-L1 Abs.

**Fig 4:**
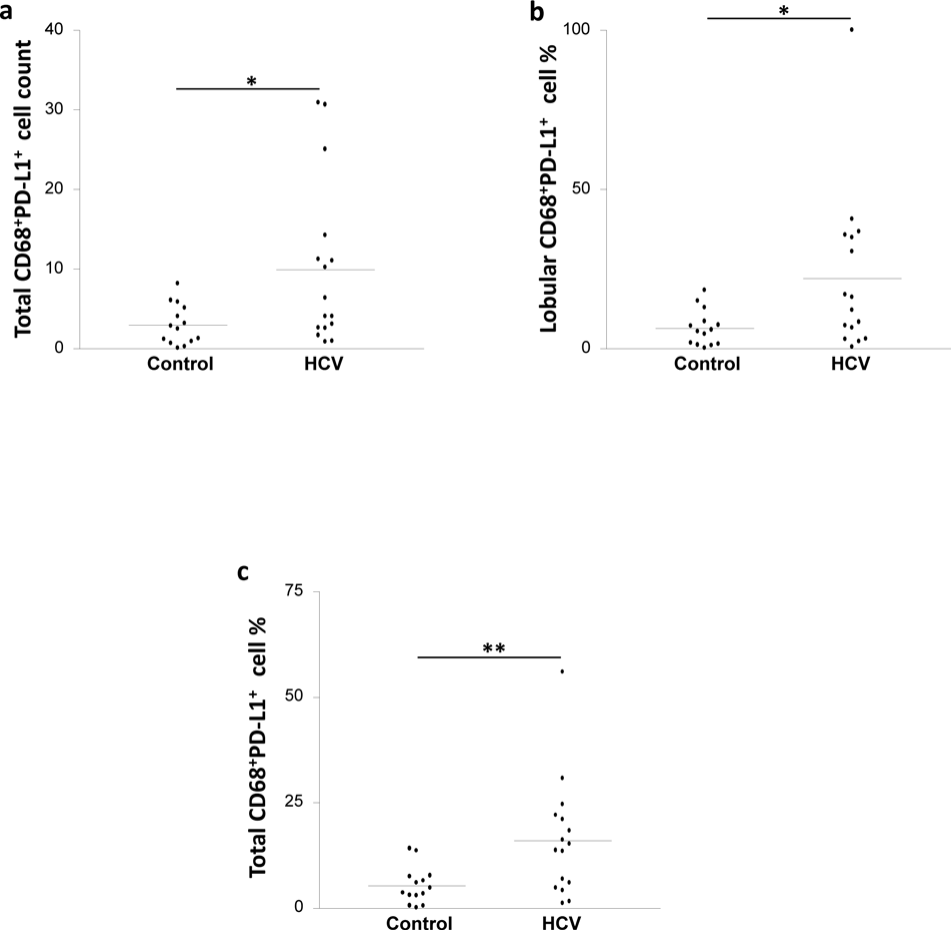
HCV infection is associated with elevated CD68^+^PD-L1^+^ cell frequency in the liver. Biopsies from HCV-infected and control individuals were stained with mouse anti-human CD68 and rabbit anti-human PD-L1 Abs. **A.** CD68^+^PD-L1^+^ cell count in the total (lobular and portal) areas of the liver of HCV-infected and control individuals. CD68^+^PD-L1^+^ cell percentage in (**B**) the lobular areas and (**C**) the total (lobular and portal) areas of the liver of control and HCV-infected individuals. * *P* value<0.05. ** *P* value<0.01.

The CD68^+^PD-L1^+^ cell counts in both the lobular and the portal areas were not significantly different between the HCV-infected patients and the controls (Table 2). However, the total CD68^+^PD-L1^+^ cell count in the HCV-infected patients was 3-fold higher than the controls (*p* = 0.028; Fig 4A). Moreover, the frequencies of CD68^+^PD-L1^+^ cells in the lobular and total areas were significantly higher in the HCV-infected patients compared with the controls [Fig 4B (*p* = 0.031) and Fig 4C (*p* = 0.007) respectively], while the frequencies of these cells in the portal areas were similar between the two groups (Table 3).

Of note, the count and frequency of CD68^+^ cells expressing CD80, CD86 or PD-L1 were neither correlated with HCV viral load (data not shown), nor associated with the age and sex of the individuals, in the HCV-infected or control group (data not shown).

### A Higher frequency of CD68^+^CD80^+^ cells compared with CD68^+^CD86^+^ and CD68^+^PD-L1^+^ cell frequencies in the lobular areas of the liver of HCV-infected patients

We further compared the expression of CD80, CD86 and PD-L1 on CD68^+^ cells within each group of the donors.

**Fig 5:**
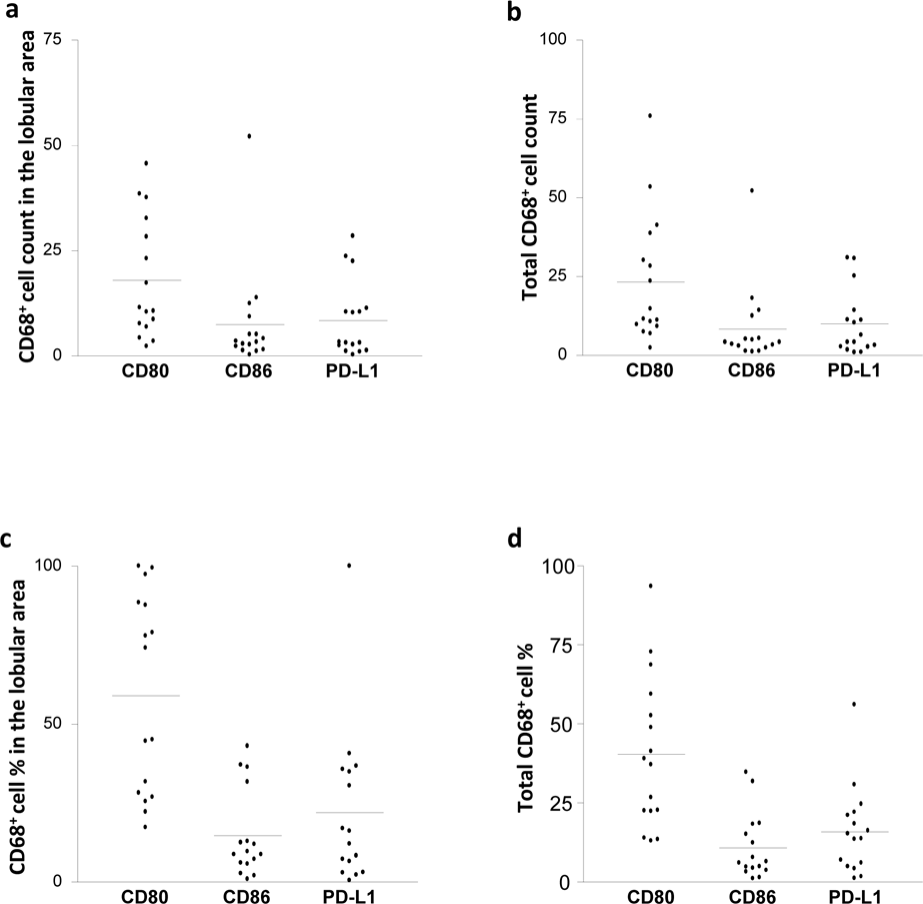
HCV infection is associated with higher CD68^+^CD80^+^ cell frequency than that of CD68^+^CD86^+^ and CD68^+^PD-L1^+^ cells in the lobular areas of the liver. Biopsies from HCV-infected individuals were stained with mouse anti-human CD68 and rabbit anti-human CD80, CD86 or PD-L1 Abs. The count of CD68^+^CD80^+^, CD68^+^CD86^+^ and CD68^+^PD-L1^+^ cells in the lobular (**A**) and the total (lobular and portal; **B**) areas are shown. The percentage of CD68^+^CD80^+^, CD68^+^CD86^+^ and CD68^+^PD-L1^+^ cell percentage in (**C**) the lobular and (**D**) the total (lobular and portal) areas are shown.

In the control group, the number and the percentages of CD68^+^CD80^+^, CD68^+^CD86^+^ and CD68^+^PD-L1^+^ cells were not different from each other in the three areas of the liver (Tables 2 and 3). In contrast, in the HCV-infected patients, we found that the count of CD68^+^CD80^+^ KCs was higher than the counts of CD68^+^CD86^+^ KCs (2.5-fold) and CD68^+^PD-L1^+^ KCs (2.1-fold) in the lobular areas (Fig 5A). There was no difference in the counts when comparing CD68^+^CD80^+^ cells with CD68^+^CD86^+^ cells or CD68^+^PD-L1^+^ cells in the portal areas, and when comparing CD68^+^CD86^+^ cells with CD68^+^PD-L1^+^ cells in the lobular and portal areas (Table 2). When the total counts were considered, the total CD68^+^CD80^+^ cell count was 2-fold higher than the total CD68^+^CD86^+^ cells and CD68^+^PD-L1^+^ cells (Fig 5B). However, there was no difference between the total counts of CD68^+^CD86^+^ and CD68^+^PD-L1^+^ cells in HCV-infected patients (Table 2).

Furthermore, the percentage of CD68^+^CD80^+^ cells was higher than that of CD68^+^CD86^+^ cells and CD68^+^PD-L1^+^ cells (4-fold and 2.6-fold respectively; Fig 5C) in the lobular areas in the HCV-infected patients. No difference was found between the percentage of CD68^+^CD80^+^ cells and CD68^+^CD86^+^ or CD68^+^PD-L1^+^ cells in the portal areas and no difference was found between the percentages of CD68^+^CD86^+^ and CD68^+^PD-L1^+^ cells in both the lobular and portal areas of the HCV-infected liver. However, the total percentage of CD68^+^CD80^+^ cells was higher than the percentage of CD68^+^CD86^+^ cells (4-fold) and CD68^+^PD-L1^+^ cells (2.5-fold; Fig 5D). No difference was found between the total percentages of CD68^+^CD86^+^ cells and CD68^+^PD-L1^+^ cells (Table 3).

### Higher percentages of CD68^+^CD80^+^ and CD68^+^CD86^+^ cells in the lobular areas compared with the portal areas of the liver in the HCV-infected patients

**Fig 6:**
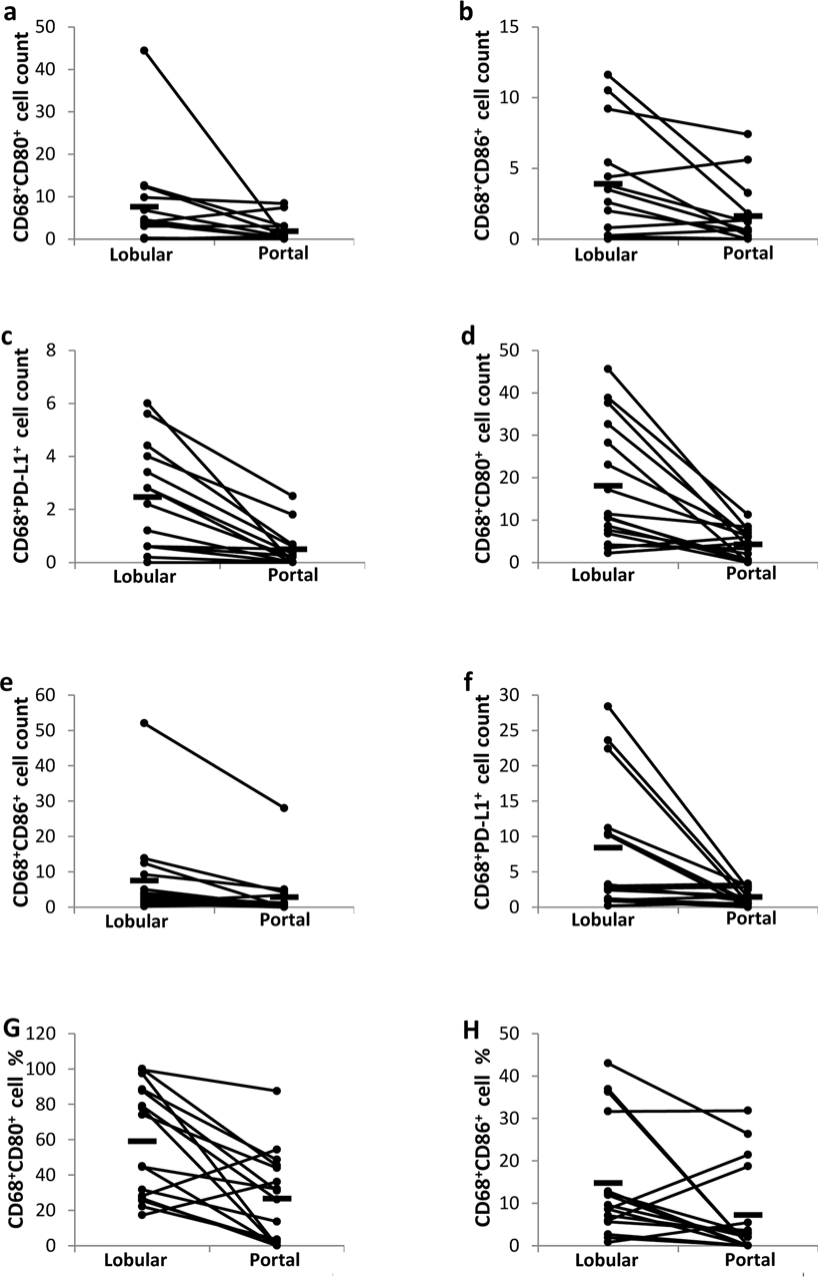
HCV infection is associated with higher CD68^+^CD80^+^ and CD68^+^CD86^+^ cell frequency in the lobular areas when compared to the portal areas of the liver. Biopsies from HCV-infected and control individuals were stained as described in the methods. (**A**) CD68^+^CD80^+^, (**B**) CD68^+^CD86^+^ and (**C**) CD68^+^PD-L1^+^ cell count in the lobular and portal areas of the liver of control individuals. (**D**) CD68^+^CD80^+^, (**E**) CD68^+^CD86^+^ and (**F**) CD68^+^PD-L1^+^ cell count and (**G**) CD68^+^CD80^+^ and (**H**) CD68^+^CD86^+^ cell percentage in the lobular and portal areas of the liver of HCV-infected patients.

In order to assess any difference in the stimulatory profile of CD68^+^ cells between the portal and the lobular areas, the counts and percentages of CD68^+^CD80^+^, CD68^+^CD86^+^ and CD68^+^PD-L1^+^ cells were compared between the two areas. In the control group, we observed higher counts of CD68^+^CD80^+^ (4-fold increase), CD68^+^CD86^+^ (2-fold increase) and CD68^+^PD-L1^+^ cells (5-fold increase) in the lobular areas compared with the portal areas (Fig 6A, 6B and 6C respectively). However, the percentages of these cells were similar in the lobular and portal areas of the liver (Table 3).

Similarly, in the HCV-infected patients, we observed higher counts of CD68^+^CD80^+^ (5-fold increase), CD68^+^CD86^+^ (3-fold increase) and CD68^+^PD-L1^+^ cells (6-fold increase) in the lobular areas compared with the portal areas (Fig 6D, 6E and 6F respectively). Interestingly, we also observed a higher percentages of CD68^+^CD80^+^ (2-fold increase) and CD68^+^CD86^+^ cells (2-fold increase) in the lobular areas compared with those in the portal areas in contrast to the control group (Fig 6G and 6H respectively). Nevertheless, the percentage of CD68^+^PD-L1^+^ cells in the lobular areas was similar to that in the portal areas of the liver (Table 3).

## Discussion

Our results showed that the CD68^+^ KC count decreased in the lobular areas of the liver during chronic HCV infection. This might be due to the programmed death of these cells during HCV infection. Apoptosis can be induced in macrophages by multiple factors. In fact, the levels of lipopolysaccharide (LPS), which can induce apoptosis in macrophages[18], are increased in the serum of HCV-infected patients, and this increase has been found to be associated with the outcome of the disease and the progression to end-stage liver disease[19,20]. Moreover, HCV infection is associated with increased IL-10 production and increased IL-10 levels in the serum[21,22]. IL-10 may induce macrophage apoptosis[23] and cause functional impairment of monocytes in HCV-infected patients[24]. This in turn may impact monocytes capacity to infiltrate the liver and differentiate to macrophages. Alternatively, microparticles, which are small membrane vesicles released from apoptotic cells, can induce apoptosis in macrophages[25,26,27]. Microparticles might be released from apoptotic hepatocytes during HCV infection [28] and cause apoptosis of macrophages. However, the exact cause of the decrease in CD68^+^ cells needs further investigation. The current knowledge about the frequency of monocytes/macrophages and KCs in the liver during HCV infection is limited and the results are controversial. One study found that the density of intrahepatic macrophages did not change in the portal areas but increased in the lobular areas in HCV-infected patients[29]. In their study, the cells were identified as CD163^+^ using immunofluorescence staining. However, in contrast to CD68, CD163 expression may be altered by stimulation[15]. Thus, identification of macrophages based on CD163 expression may be skewed since these cells are not stimulated in healthy individuals as they are in patients with infections such as HCV infection, which causes activation of the immune system. Another study found that the density of CD68^+^ cells in the portal areas but not the lobular areas was increased in chronic HCV infection[16]. The difference in the findings between the study of McGuiness et al. [16] and our present study might be due to the higher number of individuals in the control group in our study.

We observed that HCV infection did not affect the distribution of CD68^+^ cells between the lobular and the portal areas of the liver. The majority of CD68^+^ cells was found within the lobular areas, specifically within the sinusoids, which might be a consequence of transendothelial migration (TEM). This result supports previous observations showing the presence of the majority of KCs within the sinusoids in lobule of the liver[13]. However, another study, using CD163 as a marker, found that the portal tracts were more densely populated with macrophages than the lobules[29].

To our knowledge, this is the first study that assessed CD80, CD86 and PD-L1 expression in the liver of patients with HCV infection using IHC-double staining method. Interestingly, the frequency of CD68^+^ cells expressing CD80 was higher in the HCV-infected livers than the non-infected livers. However, CD86 expression did not increase in HCV-infected patients. Our finding supports the results of a study showing that the major population of monocytes/macrophages and KCs, identified based on their morphology, in HCV-infected patients was expressing CD80[13]. No previous study has investigated the expression of CD86. Furthermore, we found that in the HCV-infected patients, CD68^+^ cells in the lobular areas were more activated than those in the portal areas. In fact, the percentage of cells expressing CD80 or CD86 was higher in the lobular areas of the HCV-infected donors. Although the counts of CD68^+^CD80^+^ and CD68^+^CD86^+^ cells were higher in the lobular areas than the portal areas in both the HCV-infected and the control donors, this does not indicate a higher activation of CD68^+^ cells in the lobular areas in the control group as the total CD68^+^ cell count was also higher in the lobular areas compared to the portal areas in these donors. The percentage of activated cells (CD68^+^CD80^+^ and CD68^+^CD86^+^ cells) was similar between both areas in the control group.

Besides the co-stimulatory markers, the expression of the inhibitory maker, PD-L1, on CD68^+^ cells was increased in the HCV-infected patients. These findings are in agreement with a previous study reporting that PD-L1 expression was increased on monocytes/macrophages and KCs, identified based on their morphology, in HCV-infected livers[14].

Furthermore, our results showed for the first time that the frequency of CD68^+^CD80^+^ cells in the HCV-infected liver was higher than the frequency of CD68^+^CD86^+^ and CD68^+^PD-L1^+^ cells. Interestingly, in the control group the frequency of the expression of these three molecules was comparable. These results indicate that monocytes/macrophages and KCs in the liver of HCV-infected patients have an activated profile; however this activation fails to induce the upregulation of CD86 expression. Interestingly, HCV core antigen (Ag) expression in the liver has been found near CD80^+^ cells, and the distribution pattern of the CD80^+^ cells in the liver was very similar to that of HCV core Ag[30]. This suggests that HCV particles can be responsible for the activation of CD68^+^ cells as HCV has multiple pathogen associated molecular patterns (PAMPs) that induce APC activation[31]. While the sequences of CD80 and CD86 have about 25% similarity, both molecules can interact with CD28 and CTLA-4[7]. Whereas CD86 expression is more abundant and more quickly upregulated upon stimulation, CD80 is not commonly expressed on resting APCs, and its increase is slow following activation[7]. Therefore, CD86 might be the main ligand for CD28 early during the activation of T cells [7]. CD80 is then increased on APCs when CTLA-4 is upregulated on T cells because of their activation. CD80 has a greater ability than CD86 to induce the inhibition of T cell responses through its interaction with CTLA-4[6,7,8]. Moreover, CTLA-4 is able to strongly deplete CD80 from the surface of APCs, rendering CD80-CD28 interaction and the consequent induction of a stimulatory signal difficult[9,10]. Furthermore, CD80 is a crucial factor in the induction of regulatory T cells (T-regs)[32]. Therefore, CD86 may initiate activator responses, whereas CD80 probably controls the consequent responses[7]. Consequently, the relative expression levels of CD80 and CD86 on APCs could dictate the balance between the CD28- and CTLA-4-dependent outcomes. Thus, our results, showing the upregulation of CD80 and not CD86, are in line with the presence of a delay in the initiation of the adaptive immune response during HCV infection[3,4]. Interestingly, the liver of HCV-infected patients has a strong immunosuppressive milieu, which is associated with abundant infiltrating T-regs and upregulation of CTLA-4[33,34]. Furthermore, CTLA-4 expression is upregulated preferentially in PD-1^+^ T cells in the liver of HCV-infected patients, and these two molecules act in a synergistic way to enhance T cell exhaustion[35]. Thus, the upregulation of CD80 only with no change in CD86 levels might be an evasion strategy of HCV, and the increase of PD-L1 and CD80 but not CD86, as described in our results, might lead CD68^+^ cells in the liver of HCV-infected patients to induce the exhaustion of CTLA-4^+^ and PD-1^+^ T cells.

Altogether, the decrease in the count of CD68^+^ cells in the lobular areas of the liver and the increased expression of CD80 and PD-L1 but not CD86 observed in our study, suggest that CD68^+^ cells have a potential inhibitory profile during HCV infection. This might provide an explanation for the delayed T cell response during HCV infection and the persistence of the infection. Our findings also highlight the importance of strategies aiming to block the CD80/CTLA-4 and PD-1/PD-L1 pathways and induce optimal CD86 expression in liver CD68^+^ cells when designing new vaccinal and therapeutic anti-HCV approaches.
